# Immortalized pathological human myoblasts: towards a universal tool for the study of neuromuscular disorders

**DOI:** 10.1186/2044-5040-1-34

**Published:** 2011-11-01

**Authors:** Kamel Mamchaoui, Capucine Trollet, Anne Bigot, Elisa Negroni, Soraya Chaouch, Annie Wolff, Prashanth K Kandalla, Solenne Marie, James Di Santo, Jean Lacau St Guily, Francesco Muntoni, Jihee Kim, Susanne Philippi, Simone Spuler, Nicolas Levy, Sergiu C Blumen, Thomas Voit, Woodring E Wright, Ahmed Aamiri, Gillian Butler-Browne, Vincent Mouly

**Affiliations:** 1 Thérapie des maladies du muscle strié, Institut de Myologie, UM76, UPMC Université Paris 6, Paris, France; 2 INSERM U974, Paris, France; 3 CNRS UMR 7215, Paris, France; 4 Innate Immunity Unit, INSERM U 668, Institut Pasteur, Paris, France; 5 Service d'Oto-Rhino-Laryngologie et de Chirurgie Cervico-Faciale, Faculté de Médecine St Antoine, Université Pierre et Marie Curie, Hôpital Tenon, Paris, France; 6 The Dubowitz Neuromuscular Centre, Institute of Child Health, University College, London, UK; 7 Muscle Research Unit, Experimental and Clinical Research Center, Charité University Hospital and Max Delbrück Center for Molecular Medicine, Berlin, Germany; 8 Faculté de Médecine de Marseille, Université de la Méditerranée, Inserm UMRS 910 Génétique Médicale et Génomique Fonctionnelle, Marseille, France; 9 Department of Neurology, Hillel Yaffe Medical Center, PO Box 169, Hadera, 38100, Israel; 10 UT Southwestern Medical Center, Department of Cell Biology, Dallas, TX 75390, USA; 11 Laboratoire LBCM, Departement de Biologie, Faculté des Sciences, Agadir, Maroc

## Abstract

**Background:**

Investigations into both the pathophysiology and therapeutic targets in muscle dystrophies have been hampered by the limited proliferative capacity of human myoblasts. Isolation of reliable and stable immortalized cell lines from patient biopsies is a powerful tool for investigating pathological mechanisms, including those associated with muscle aging, and for developing innovative gene-based, cell-based or pharmacological biotherapies.

**Methods:**

Using transduction with both telomerase-expressing and cyclin-dependent kinase 4-expressing vectors, we were able to generate a battery of immortalized human muscle stem-cell lines from patients with various neuromuscular disorders.

**Results:**

The immortalized human cell lines from patients with Duchenne muscular dystrophy, facioscapulohumeral muscular dystrophy, oculopharyngeal muscular dystrophy, congenital muscular dystrophy, and limb-girdle muscular dystrophy type 2B had greatly increased proliferative capacity, and maintained their potential to differentiate both *in vitro *and *in vivo *after transplantation into regenerating muscle of immunodeficient mice.

**Conclusions:**

Dystrophic cellular models are required as a supplement to animal models to assess cellular mechanisms, such as signaling defects, or to perform high-throughput screening for therapeutic molecules. These investigations have been conducted for many years on cells derived from animals, and would greatly benefit from having human cell models with prolonged proliferative capacity. Furthermore, the possibility to assess *in vivo *the regenerative capacity of these cells extends their potential use. The innovative cellular tools derived from several different neuromuscular diseases as described in this report will allow investigation of the pathophysiology of these disorders and assessment of new therapeutic strategies.

## Background

Muscular dystrophies constitute a heterogeneous group of genetic muscle diseases characterized by progressive muscle weakness, wasting and degeneration, some of these features are common to muscle aging [1,2]. Over the past few years, the genetics and pathophysiology of some of these diseases has been deciphered, stimulating the development of novel gene-based (or mRNA-based) (for example, gene therapy, exon-skipping or codon read-through), cell-based and pharmacological therapies [3], which can either target the mutation directly, or target the consequences of that mutation, such as muscle wasting, atrophy or denervation. To assess these rapidly developing therapeutic advances, there is a crucial need to develop standardized tools to determine the cellular and molecular mechanisms that trigger the physiopathologic modifications, and to assess these new therapeutic strategies in preclinical trials. Transgenic mice have often been used to investigate the physiopathology of muscular dystrophies [4-6]; however, the mutation remains in a murine context, and there are often major differences between humans and mice; for example, a mutation in the dystrophin gene results in a mild pathological phenotype in *mdx *mice but in a progressive and fatal disease (Duchenne muscular dystrophy; DMD) in humans. Furthermore, not every mutation can be created and evaluated in murine models, and mechanisms common to aging and dystrophies may differ between mice and humans. Consequently, human primary myoblasts isolated from dystrophic patient biopsies provide the most pertinent experimental models to assess a variety of human genetic mutations in their natural genomic environment. Although *in vitro *models do not fully recapitulate the *in vivo *environment, cell-culture systems allow rapid, high-throughput screening of molecules or oligonucleotides, and new strategies can be easily tested prior to validation in animal models, which is a costly and time-consuming process. The main drawbacks of using *in vitro *primary cultures of human cells derived from muscle biopsies are their purity, their limited proliferative capacity, and the variation in phenotype when amplified *in vitro*; their phenotype will always be confounded by modifications due to cellular senescence, which will progressively occur during cell amplification [7,8].

The two major mechanisms responsible for this replicative cellular senescence seen in human myoblasts are (i) activation of the p16-mediated cellular stress pathway, and (ii) the progressive erosion of telomeres at each cell division until they reach a critical length that will trigger p53 activation and cell-cycle exit [9,10]. Introduction of the telomerase catalytic subunit (*human telomerase reverse transcriptase*; *hTERT*) cDNA alone will result in an extension of the lifespan and even immortalization in a variety of cell types, including endothelial cells and fibroblasts [11,12]. However, we have shown that the expression of both *hTERT *and *cyclin-dependent kinase (CDK)-4 *is required to successfully overcome cellular senescence in human myoblasts [13]; while hTERT elongates the telomere, CDK-4 blocks the p16^INK4a^-dependent stress pathway.

In the present study, our goal was to create a large collection of immortalized human myoblasts isolated from a wide range of neuromuscular disorders (DMD, facioscapulohumeral muscular dystrophy (FSHD), oculopharyngeal muscular dystrophy (OPMD), limb-girdle muscular dystrophy (LGMD2B or dysferlinopathy) and congenital muscular dystrophy (CMD)), which could be used as experimental tools to study these diseases and to develop new therapeutic strategies.

DMD is the most common childhood muscular dystrophy. It is caused by mutations in the dystrophin gene encoding an essential protein of the muscle membrane cytoskeleton [14], leading to rapid and progressive skeletal-muscle weakness. FSHD is a progressive muscle disease caused by contractions in a 3.3 kb repeat region (D4Z4) located at 4q35.2 [15], which first affects the muscles of the face and upper limb girdle with asymmetry, and later the lower limb girdle. OPMD is a rare, autosomal dominant, late-onset degenerative muscle disorder caused by a short (GCG)_n _triplet expansion in the *poly(A) binding protein nuclear 1 *(*PABPN1*) gene [16], which affects the eyelid and pharyngeal muscles. LGMD2B is a recessive muscle disease caused by mutations in the dysferlin gene, a muscle membrane protein known to be involved in membrane repair [17] and trafficking. The disease is characterized by early and slowly progressive weakness and atrophy of the pelvic and shoulder girdle muscles in early adulthood. Finally, CMD refers to a clinically and genetically heterogeneous group of dystrophies, which result in the onset of muscle weakness at birth or in childhood, and involve mutations in several proteins such as collagen, laminin, integrin, and nesprin 1 [18].

In this study, we report for the first time that for each of these muscular dystrophies, we were able to produce reliable and stable immortalized cell lines from human myoblasts isolated from biopsies, resulting in robust *in vitro *models that can also be implanted *in vivo*. This non-exhaustive list of cellular models will provide powerful and valuable tools for the scientific community investigating these pathological conditions and/or their mechanisms. as they overcome the problem of limited proliferation usually present in myoblasts. These models should also be useful in the development of gene or cell therapies and pharmacological strategies for muscular dystrophies, some of which might also be used to combat muscle weakness in the elderly.

## Methods

### Ethics approval

Muscle biopsies (Table 1) were obtained from the BTR (Bank of Tissues for Research, a partner in the EU network EuroBioBank) or from neurologists, in accordance with European recommendations and French legislation. Surgical procedures were performed in accordance with the legal regulations in France and European Union ethics guidelines for animal research.

**Table 1:** Muscle biopsies obtained from various neuromuscular dystrophies.

Name	Disease	Genetic defect	Donor muscle	Age
CTRL	None	None	Semitendinosus	25 years

CMD	Congenital muscular dystrophy	2345G > T; *nesprin-1 *gene	Paravetrebral	16 years

DMD	Duchenne muscular dystrophy	Deletion of exon 48-50; *dystrophin *gene	Quadriceps	20 months

FSHD	Fascioscapulohumeral muscular dystrophy	2 D4Z4 contraction	Subscapularis	27 years

LGMD2B	Limb-girdle muscular dystrophy type 2B	1448C > A and 107T > A; *dysferlin *gene	Quadriceps	40 years

OPMD	Oculopharyngeal muscular dystrophy	Expansion (GCG)_9_-(GCG)_6_; *PABPN1 *gene	Cricopharyngeal	60 years

### Human myoblast cultures

Human myoblasts were isolated from biopsies and cultivated as described previously [19] in a growth medium consisting of 199 medium and DMEM (Invitrogen Carlsbad, CA) in a 1:4 ratio, supplemented with 20% FCS (Invitrogen), 2.5 ng/ml hepatocyte growth factor (Invitrogen), 0.1 μmol/l dexamethasone (Sigma-Aldrich, St. Louis, MO, USA) and 50 μg/ml gentamycin (Invitrogen). The myogenic purity of the populations was monitored by immunocytochemistry using desmin as marker. Enrichment of myogenic cells was performed using an immunomagnetic cell sorting system (MACS; Miltenyi Biotec, Paris, France) according to the manufacturer's instructions. Briefly, cells were labeled with anti-CD56 (a specific marker of myoblasts) microbeads, and then separated in a MACS column placed in a magnetic field. Purification was checked by immunochemistry using a desmin marker. Differentiation was induced at confluence by replacing the growth medium with DMEM supplemented with 100 μg/ml transferrin, 10 μg/ml insulin and 50 μg/ml of gentamycin (Sigma-Aldrich).

### Cell transduction

*hTERT *and *Cdk4 *cDNA were cloned into different pBABE retroviral vectors containing puromycin and neomycin selection markers, respectively. Infection was carried out as described previously [20]. Transduced cell cultures were selected with puromycin (0.2 μg/ml) and/or neomycin (0.3 mg/ml) for 8 days. The infected cells were purified as described previously if necessary, and were then seeded at clonal density. Selected individual myogenic clones were isolated from each population, using glass cylinders, and their proliferation and differentiation capacities were characterized.

### Telomere length analysis

Genomic DNA was extracted from each proliferating cell line using a salting-out procedure. Telomere length was determined by using a quantitative (q)PCR method, as previously described [21,22]. PCR amplification was achieved using telomere (T) and single-copy gene 36B4 (acidic ribosomal phosphoprotein P0) (S) primers. The mean telomere length was calculated as the ratio of telomere repeats to 36B4 copies, represented as the T:S ratio. Each sample was run in triplicate, using 20 ng of DNA per replicate, and three independent runs were analyzed. The primer sequences and detailed PCR protocols used are available on request.

### Reverse transcriptase PCR

To analyze the expression of myogenic markers in proliferating primary and immortalized cell lines, 1 μg RNA from each cell line was used for the cDNA synthesis (Superscript III; Invitrogen) using random hexamer primers. cDNA (1 μl) was used as a template for PCR using N-CAM, MyoD and desmin specific primers. The primer sequences and detailed PCR protocols used are available on request.

### Induction of host muscle regeneration and implantation of human cells

Immunodeficient Rag2^-/- ^γC^-/- ^C5^-/- ^mice aged 2 to 3 months were anesthetized with an intraperitoneal injection of ketamine hydrochloride (80 mg/kg) and xylasin (10 mg/kg) (Sigma-Aldrich). To induce severe muscle damage and trigger regeneration, the recipient tibialis anterior (TA) muscles were exposed to cryodamage, and a single injection of immortalized human cells (15 μl of cell suspension containing 2.5 × 10^5 ^or 5 × 10^5 ^cells in PBS) was administered as described previously [23]. Four weeks after transplantation, the recipient TA muscles were dissected, mounted in gum tragacanth, and frozen in liquid nitrogen-cooled isopentane for later analysis.

### Immunofluorescence

*In vitro *and *in vivo *characterizations were performed by immunolabeling as described previously [23-25]. Antibodies used were directed against myosin isoforms (MF20, mouse IgG2b, 1:20 dilution; Developmental Studies Hybridoma Bank, DSHB, Iowa City, IA), lamin A/C (clone JOL2, mouse IgG1, 1:300; AbCam, Cambridge, Cambridgeshire, UK), lamin A/C (NCL-LAM A/C, clone 636, mouse IgG2b, 1:400, Novocastra, Newcastle-upon-Tyne, Tyne and Wear, UK), spectrin (NCL-Spec1, clone RBC2/3D5, mouse IgG2b, 1:50; Novocastra), and laminin (rabbit polyclonal, Z 0097, 1:400; Dako, Trappes, France). The secondary antibodies used were Alexa Fluor 488-conjugated goat anti-mouse IgG2b (Molecular Probes, Montluçon, France), Alexa Fluor 647-conjugated goat anti-rabbit (Molecular Probes), and Cy3-conjugated goat anti-mouse IgG1 (Jackson Immunoresearch, West Grove, PA, USA). Images were visualized using a microscope (Olympus Corp., Tokyo, Japan), and digitized using a charge-coupled device (CCD) camera (Olympus Corp., Tokyo, Japan).

### Antisense oligonucleotides transfection and reverse transcriptase PCR

Cells were seeded in six-well plates and grown in growth medium. Transfection of antisense oligonucleotides (AONs) was performed using 1 μl of transfection reagent (Lipofectamin 2000; Invitrogen) per μg of AONs for 4 hours. The chemistry used for AONs was 2'-O-methyl-phosphorothioates. All transfections were performed with at least two independent duplicates. Cells were changed to differentiation medium before transfection. Typically 24 to 48 hours after transfection, RNA was extracted from the cells using Trizol or Qiagen column kit (Qiagen Inc., Valencia, CA, USA). 1 μg RNA was used for the cDNA synthesis (Superscript III; Invitrogen) with DMD exon-specific primers. cDNA (2 μl) was used as a template for a first PCR reaction. From this first reaction of 25 cycles, 1 μl of the product was removed and used as a template for a second nested PCR of 35 cycles. PCR products were analyzed on 1.5 to 2% agarose gels. The primer sequences and detailed PCR protocols used are available on request.

## Results

### Immortalized myoblast lines generated from dystrophic muscles

**Figure 1:**
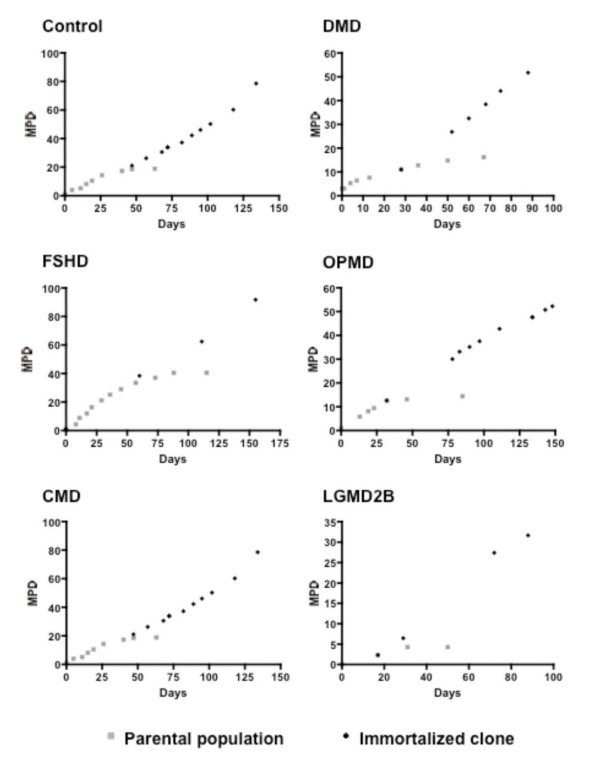
**Proliferative potential**. Lifespan plots (mean population doublings; MPD) of the parental populations and the immortalized cell lines derived from them.

Primary cultures from distinct muscular dystrophies (DMD, FSHD, OPMD, CMD and LGMD2B, Table 1) were co-transduced with two retroviral vectors expressing *hTERT *and *CDK-4 *cDNA. Co-transduced cells were selected by neomycin and puromycin and then purified using magnetic beads coupled to antibodies directed against the myogenic marker CD56. Following culture at clonal density, individual myogenic clones with extended proliferative lifespans, as compared to the untranduced cells, were isolated from each population. In contrast to the parental populations, which stopped proliferating at various stages of the culture, depending on the type of dystrophy, the selected immortalized clones were still able to proliferate after prolonged amplification *in vitro *under the same culture conditions (Figure 1). All immortalized clones were cultivated until they had achieved at least twice as many divisions as the parental population.

Telomere length was measured in each clone (Table 2) and ranged from 10.3 kb to 24.8 kb with no difference between the clones and control immortalized myoblasts (17.6 kb). The length of the telomeres in all of the immortalized myogenic clones was always well above the 6 to 7 kb limit usually seen in control cells that are reaching senescence, but remained within the range of that seen both in myoblasts and in other stem cells (12 to 20 kb).

**Table 2:** Mean telomere length of control and immortalized cell lines.

Name	Clone	Number of divisions	Telomere length, kb, mean ± SEM
CTRL	C25Cl48	127	17.6 ± 0.3

CMD	CMDCl12	42	20.8 ± 1.7

DMD	DMDCl2	57.9	10.3 ± 0.1

FSHD	FSHDCl17	37.9	24.8 ± 1.6

LGMD2B	LGMD2Cl11	27.4	17.2 ± 3.0

OPMD	OPMDCl2	47.6	20.0 ± 0.5

CMD, congenital muscular dystrophy; DMD, Duchenne muscular dystrophy; FSHD, fascioscapulohumeral muscular dystrophy; LGMD2B, limb-girdle muscular dystrophy type 2B; OPMD, oculopharyngeal muscular dystrophy

### *In vitro *characterization of immortalized cells

**Figure 2:**
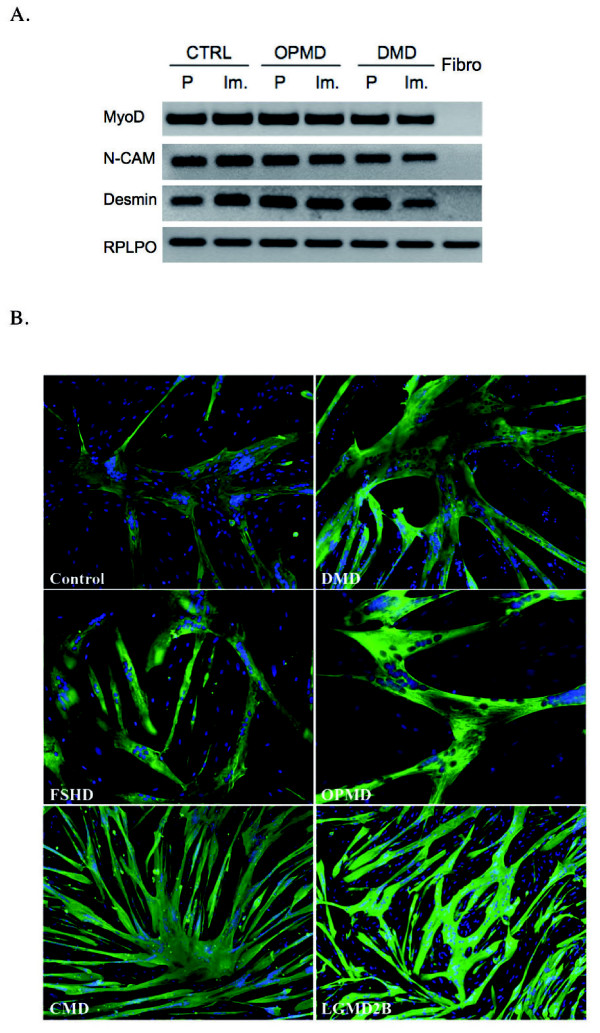
**Myogenic markers and *in vitro *differentiation**. **(A) **Reverse transcriptase PCR comparing primary (P) and immortalized (Im.) cell lines (control, oculopharyngeal muscular dystrophy (OPMD), Duchenne muscular dystrophy (DMD)) for myogenic markers (MyoD, N-CAM and desmin). Fibroblasts were used as a negative control. **(B) **Immunofluorescence was carried out using MF20, an antibody directed against sarcomeric myosin (green) after 5 days of differentiation. Specific antibody labeling was visualized using Alexa Fluor 488 secondary antibody (green). Nuclei were visualized with Hoechst (blue). Original magnification × 100.

To confirm that the immortalized cell lines maintained their myogenic signature, we compared the expression of several markers in proliferating primary and immortalized cell lines from control, OPMD and DMD biopsies. In all of them, we confirmed the expression of the myogenic markers desmin, neural cell adhesion molecule (N-CAM) and MyoD (Figure 2A).

In addition, we also tested their ability to differentiate into myotubes, using immunostaining with MF20 antibody, which recognizes all skeletal-muscle myosin heavy chains (MyHCs). After 5 days in differentiation conditions, all of the immortalized cell lines were able to fuse into myotubes expressing MyHCs (Figure 2B).

### Induction of host muscle regeneration and implantation of human cells

**Figure 3:**
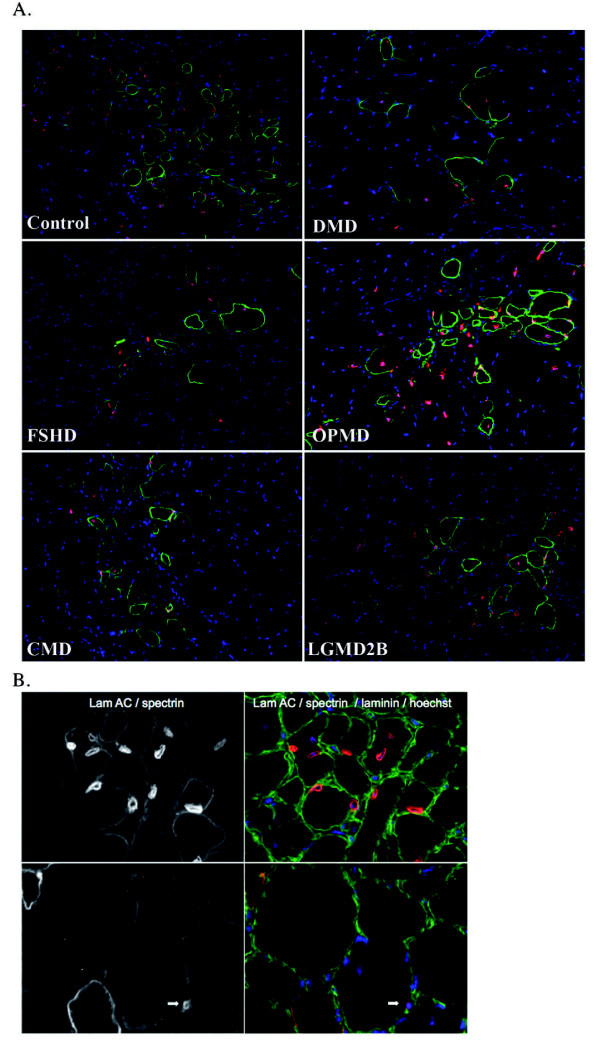
**Detection of human immortalized cells injected into in the tibialis anterior muscle of Rag2^-/- ^γC^-/- ^C5^-/- ^mice**. **(A) **Human nuclei were visualized using an anti-lamin A/C antibody (red) and fibers expressing human proteins were visualized using an anti-human spectrin-specific antibody (green). Nuclei are counterstained with Hoechst. Original magnification × 200. **(B) **The immortalized cells were present preferentially as myonuclei (top panel), with a small number found in interstitial space (arrow, bottom panel), identified by human lamin expression (laminin staining in green, laminA/C and spectrin staining in red, Hoechst in blue, on the LGMD2B muscle section as an example). Original magnification × 600.

To investigate the in vivo behavior of these immortalized cells, cells were grafted into damaged TA muscles of Rag2^-/- ^γC^-/- ^C5^-/- ^mice; injected muscles were analyzed 4 weeks after transplantation, which corresponds to a complete fiber regeneration process, using antibodies specific for human lamin A/C (expressed in all human nuclei) and human spectrin (expressed in differentiated fibers). For each injected clone (control, DMD, FSHD, OPMD, CMD or LGMD2B), mature muscle fibers containing human spectrin protein and human lamin A/C+ nuclei were seen (Figure 3A). No tumors were ever observed in these immunodeficient mice.

Using antibodies specific for the basal lamina protein laminin, lamin A/C (human nuclei) and spectrin (specific to the human protein) to identify fiber sarcolemma, we investigated if these cell lines could replenish the muscle stem-cell niche (allowing self-renewal), at the periphery of the muscle fiber and beneath the basal lamina. Whereas the vast majority of the lamin A/C-positive nuclei (97%) were found as myonuclei (upper panel, Figure 3B), we observed the unexpected finding that all the human cells outside the muscle fibers were present in the interstitial space, separated from the fibers by a basal lamina (lower panel, Figure 3B), and not in the satellite-cell niche, suggesting that the immortalized cells were engaged preferentially in the differentiation pathway and not in the self-renewal process.

### Immortalized cell lines as a useful tool for therapeutic preclinical studies

**Figure 4:**
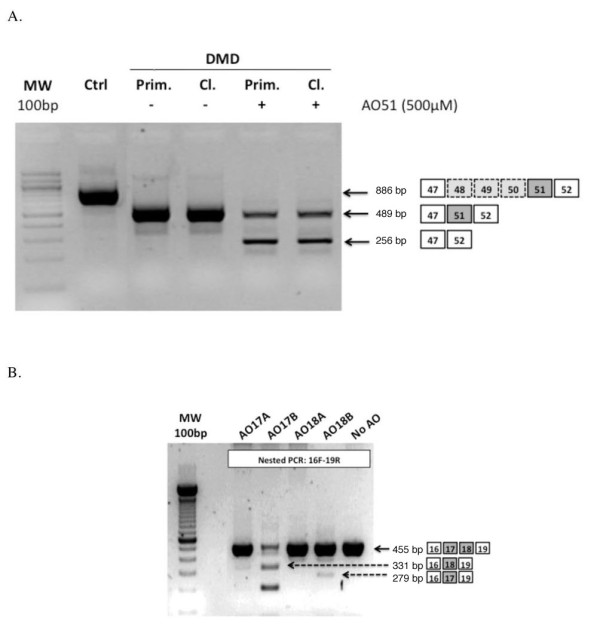
**Antisense oligonucleotides (AONs) tested in immortalized cells for exon-skipping pre-clinical studies**. **(A) **Validation of AO51 efficacy for exon 51 skipping on primary (Prim.; Duchenne muscular dystrophy (DMD)) and immortalized cultures of a Δ48-50 patient with DMD. **(B) **Evaluation of the efficacy of various AONs on control immortalized cells targeting exons 17 and 18.

To show that these cell lines could be powerful tools to develop therapeutic strategies, we used them to evaluate the efficiency of AONs in an exon-skipping strategy for DMD. The first one tested was AO51, which resulted in efficient skipping of exon 51 using both the primary and immortalized DMD cell lines (Δ48 to 50; Figure 4A) and this AON is currently being used in a phase I/II clinical trial. Using immortalized control cells, we were able to screen a range of AONs targeting exons 17, 18, 21, 22, 43, 44 and 45 of the dystrophin gene. RT-PCR identified efficient skipping for two of them (Figure 4B, and data not shown).

## Discussion

Immortal human cell lines, as long as they retain their capacity to express a specific program, are essential to study cellular and molecular mechanisms (as exemplified by the number of studies conducted on the mouse cell line C2C12), and responses to potential therapeutic strategies. The relatively short proliferative lifespan of human myoblasts, reduced even more in dystrophic conditions by successive cycles of degeneration/regeneration *in vivo *prior to isolation and the modification in their myogenic potential as they approach senescence [26-28], limits their potential use. As a consequence, any assessment of pathological mechanisms or of therapeutic strategies will be biased by the presence of senescent cells, which will modify the behavior of the population. This is even more crucial for high-throughput screening of molecules, for which large numbers of cells are required. Immortalization can solve this problem, as long as the cell lines are stable and retain most of the characteristics of the unmodified parental population. This has been shown to be a problem with the C2C12 cell line, as the phenotype drifts and therefore can vary both within and between different laboratories.

Immortal cell lines have been generated from human skeletal muscle, such as those derived from rhabdomyosarcomas, a rare form of skeletal-muscle tumor. However, these lines often have impaired fusion characteristics and perturbed myogenic programs [29,30]. Other approaches have used transduction of the large T antigen from SV40, which binds Rb and p53, but does not stop telomere shortening. Although these cells do have an extended lifespan, they are not immortal, and extensive telomeric erosion results in an increased frequency of chromosomal rearrangement [31] and a defective differentiation program [32]. More recently, cell lines were generated by the transduction of primary myoblasts with both hTERT and Bmi-1, which downregulates both the p16 and p19Arf tumor suppressor genes, encoded by the Ink4 locus. These cells had an extended lifespan with no chromosomal rearrangement, but the differentiation potential of control myoblasts was found to be impaired [33]. This year, a report described the same approach as we have used in the present study (introduction of hTERT and CDK4) using muscle cells isolated from patients affected with FSHD [34]. The FSHD mutation causes a defect within myogenic cells, thus the establishment of a clonal myogenic cell line permit reproducible study of the consequences of this mutation within myoblasts without the contamination of non-myogenic cells. The decrease in myogenicity in the primary culture, and consequently the enrichment of non-myogenic cells during amplification, is a considerable problem in the study of dystrophy diseases. For example, in 2006, we described rapid loss of myogenicity during successive passages of primary cultures of muscle cells isolated from patients with OPMD [35]; many muscle diseases are subject to a similar decrease in myogenicity. In this report, we describe the isolation of immortalized human myoblast lines from a wide range of neuromuscular diseases, using a combination of hTERT and CDK-4. This wide range of diseases paves the way to searching for common mechanisms between distinct dystrophies, and even those shared with muscle aging, as opposed to those mechanisms specific for each disease. We found that these cell lines have extended proliferative lifespans, and maintain their capacity to differentiate both *in vitro *and *in vivo *after transplantation into the regenerating muscles of immunodeficient mice. We found that these human myoblast cell lines expressing myogenic markers could colonize the host muscles and form mature fibers, thus providing an ideal model to assess therapeutic strategies *in vivo*, which is closer to *bona fide *differentiation of muscle stem cells than the converted fibroblasts described previously [36]. We also found that these cells do not replenish the muscle stem-cell niche as primary human myoblasts do under the same conditions of implantation [23], but are engaged primarily in the differentiation pathway. The process of immortalization involves overexpression of both hTERT and CDK-4, and further investigations will be needed to analyze how this may influence the balance between self-renewal and differentiation.

## Conclusions

These human myoblast lines represent a powerful tool to assess signaling and/or functional deregulations in neuromuscular diseases, particularly those in which these mechanisms have not yet been clearly elucidated (FSHD, OPMD) or those with features common to muscle aging, such as atrophy or muscle wasting. We have described only a subset of the cell lines produced; we have now generated more than 35 cell lines with various mutations covering a range of 14 different pathologies, as well as cell lines from control subjects of various ages. The *in vivo *implantation of these cells offers the possibility to investigate the consequences of defined mutations on cellular behavior *in vivo*, particularly with regard to their regenerative capacity. Finally, the development of therapeutic strategies, whether these strategies imply gene, cell or pharmacological therapy involving high-throughput screening, is facilitated using these tools before assessment in animal models. As an example, we have described a rapid screen of a range of AONs in an exon-skipping strategy for DMD. In conclusion, the co-transduction of hTERT and CDK4 generates human myoblasts immortal in culture that maintain myogenic potential both *in vitro *and *in vivo*, offering new cell paradigms for pathophysiological studies and novel therapeutic strategies.

## List of abbreviations

AONs: antisense oligonucleotides; CDK-4: cyclin-dependent kinase 4; CMD: congenital muscular dystrophy; DMD: Duchenne muscular dystrophy; DMEM: Dulbecco's modified Eagle's medium; FCS: fetal calf serum; FSHD: facioscapulohumeral muscular dystrophy; hTERT: human telomerase reverse transcriptase; LGMD2B: limb-girdle muscular dystrophy type 2b; N-CAM: neural cell adhesion molecule; OPMD: oculopharyngeal muscular dystrophy; PBS: phosphate-buffered serum; PCR: polymerase chain reaction.

## Competing interests

The authors declare that they have no competing interests.

## Authors' contributions

KM, CT, AB, EN, and SC designed and performed the *in vitro *and *in vivo *experiments, analyzed data, and wrote the manuscript. AW, PKK, SM, JK, and AA provided technical support. JDS provided the immunodeficient Rag2^-/- ^γC^-/- ^C5^-/- ^mice for the *in vivo *experiments. JLSG, FM, SP, SS, NL, SB, and TV provided biopsies, and WEW provided DNA constructs. TV and AA discussed the results and gave expert advice. GBB and VM provided conceptual input and supervision. and wrote the manuscript. All authors read and approved the final manuscript.
